# Exercise Attenuates PCB-Induced Changes in the Mouse Gut Microbiome

**DOI:** 10.1289/ehp.1306534

**Published:** 2013-04-26

**Authors:** Jeong June Choi, Sung Yong Eum, Evadnie Rampersaud, Sylvia Daunert, Maria T. Abreu, Michal Toborek

**Affiliations:** 1 Department of Biochemistry and Molecular Biology,; 2 John P. Hussman Institute for Human Genomics, and; 3 Division of Gastroenterology, Department of Medicine, University of Miami School of Medicine, Miami, Florida, USA

**Keywords:** environmental toxicants, exercise, gut microbiome, polychlorinated biphenyls, PhyloChip

## Abstract

Background: The gut microbiome, a dynamic bacterial community that interacts with the host, is integral to human health because it regulates energy metabolism and immune functions. The gut microbiome may also play a role in risks from environmental toxicants.

Objectives: We investigated the effects of polychlorinated biphenyls (PCBs) and exercise on the composition and structure of the gut microbiome in mice.

Methods: After mice exercised voluntarily for 5 weeks, they were treated by oral gavage with a mixture of environmentally relevant PCB congeners (PCB153, PCB138, and PCB180; total PCB dose, 150 µmol/kg) for 2 days. We then assessed the microbiome by determination of 16S rRNA using microarray analysis.

Results: Oral exposure to PCBs significantly altered the abundance of the gut microbiome in mice primarily by decreasing the levels of Proteobacteria. The activity level of the mice correlated with a substantial shift in abundance, biodiversity, and composition of the microbiome. Importantly, exercise attenuated PCB-induced changes in the gut microbiome.

Conclusions: Our results show that oral exposure to PCBs can induce substantial changes in the gut microbiome, which may then influence their systemic toxicity. These changes can be attenuated by behavioral factors, such as voluntary exercise.

The gut microbiome is a collection of bacteria that resides in the host gastrointestinal tract. As many as 10^14^ microbes are in the human gut (Ley et al. 2006), accounting for 15,000–36,000 species of bacteria (Frank et al. 2007). The main bacterial phyla comprising the human gut microbiome are the gram-negative Bacteroidetes and Proteobacteria and the gram-positive Actinobacteria and Firmicutes (Frank et al. 2007). Each host has a unique composition of gut microbiota, which implies highly individual responses to environmental stressors and suggests a role for gut microbiota in future personalized health strategies (Kinross et al. 2011).

Recent evidence has implicated the gut microbiome in the development of a wide range of disorders, including obesity, diabetes, metabolic dysfunctions, vascular disease, and inflammatory bowel disease (Kinross et al. 2011). Strong evidence also indicates the critical role of the gut microbiota in drug metabolism and toxicity, energy metabolism, immune functions, and postsurgical recovery (Holmes et al. 2011; Kinross et al. 2011; Tilg and Kaser 2011). Moreover, the gut microbiome has been reported to regulate psychiatric health and influence etiopathology of autism. For example, Bravo et al. (2011) reported that chronic administration of *Lactobacillus rhamnosus* induced anxiolytic and antidepressant effects by modulating the expression of GABA receptors in the brain, and Lyte et al. (2006) observed that infection with *Citrobacter rodentium* induced anxiety-like behaviors via vagal sensory regulation.

Despite these diverse effects on human health, the influence of the microbiome on the toxicity of environmental pollutants and its role in disease risk are largely unknown (Betts 2011). It was recently suggested that preabsorptive metabolism can modify toxicity of environmental pollutants, influencing their health effects (Lapanje et al. 2007; Pinyayev et al. 2011). The most compelling evidence illustrating this phenomenon was obtained in studies on biotransformation of heavy metals by the gut microbiome. For example, Pinyayev et al. (2011) reported that anaerobic microbiota of mouse cecum converts arsenate into oxyarsenicals and thioarsenicals; Lapanje et al. (2007) observed that exposure to mercury altered the bacterial community in the gut of a terrestrial isopod (*Porcellio scaber*); and Liebert et al. (1997) found that gram-negative fecal bacteria were involved in the biotransformation of mercury. Experiments on germ-free mice also provided evidence that the gut microbiome can regulate the expression of cytochrome P450 enzymes, which are involved in the metabolism of a variety of xenobiotics, including environmental chemicals (Claus et al. 2011). Indeed, human colon microbiota can transform polycyclic aromatic hydrocarbons (PAHs) to estrogenic metabolites (Van de Wiele et al. 2005). These findings are significant because PAH toxicity has been linked to estrogenicity of the compounds, thus suggesting that PAH bioactivation in the colon should be taken into consideration when assessing risk (Van de Wiele et al. 2005). In addition, the role of gut microbiota, as well as its variability in relation to the disposition of environmental chemicals in the human body and its contribution to the development of obesity and diabetes, have recently been recognized (Snedeker and Hay 2012).

The present study was designed on the basis of recent evidence implicating the role of the gut microbiome in risks associated with exposure to environmental chemicals (Betts 2011). We examined whether exposure to polychlorinated biphenyls (PCBs) could affect the abundance and composition of the gut microbiome. There is growing interest in the role of behavioral factors in modulating toxicity of environmental pollutants; although the role of nutrition has been explored (e.g., Majkova et al. 2008), the impact of exercise on the health effects of toxicants is unknown. Exercise can influence the outcomes of disorders associated with alterations in the gut microbiome (Martin 2011); therefore, we hypothesized that physical activity may affect the composition of the gut microbiota and thus influence the impact of environmental toxicants. Our results indicate that PCBs can induce profound changes in the microbial composition of the gut and that exercise can attenuate these PCB-induced effects on the intestinal microbiome.

## Materials and Methods

*Animals and voluntary exercise*. All animal protocols were approved by the Institutional Committee on Animal Care of the University of Miami School of Medicine and followed National Institutes of Health guidelines (National Research Council 2011). The animals were treated humanely and with regard for alleviation of suffering. Male C57BL/6 mice (11–13 months of age; Charles River Laboratories, Wilmington, MA) were housed under 12:12-hr light/dark conditions with access to food and water *ad libitum*. Mice were randomly assigned to either the exercised or sedentary group (*n* = 6/group). Mice were housed individually in plastic cages equipped with a running wheel (Coulbourn Instruments, Whitehall, PA); however, wheels were locked in cages of sedentary mice. Running activity was monitored 24 hr/day, 7 days/week and analyzed using Clocklab (Actimetrics, Wilmette, IL) and Matlab (Mathworks, Natick, MA) software. Mice were allowed to exercise for 5 weeks, including 1 week to adapt to running and solitary housing.

Mice averaged 10–12 km/24 hr. The average speed was 1.12 km/hr, and the mean ± SE time spent running was 10.3 ± 0.33 hr/day. Exercised mice had 30% lower body weight on average than sedentary mice. At the end of the exercise period, we observed no changes in gut transit or stool frequency between the two groups.

*PCB exposure*. We selected the type of PCB congeners and their relative ratio based on PCB content in contaminated food (Ashley et al. 2000). The three environmentally relevant PCB congeners, PCB138, PCB153, and PCB180 (AccuStandard, New Haven, CT), were mixed at a molar ratio of 1.7:3.2:1 in tocopherol-stripped safflower oil (vehicle; Dyets Inc., Bethlehem, PA). The total PCB dose of 150 μmol/kg administered to mice results in a PCB plasma level of 5 μM (Choi YJ et al. 2010), which is comparable to plasma levels of PCBs in an acutely exposed human population (Jensen 1989; Wassermann et al. 1979). All treatments were performed via oral gavage using an 18-G gavage needle (3 cm length, curved, 2.25 mm ball diameter; Popper and Sons, New Hyde Park, NY).

**Figure 1:**
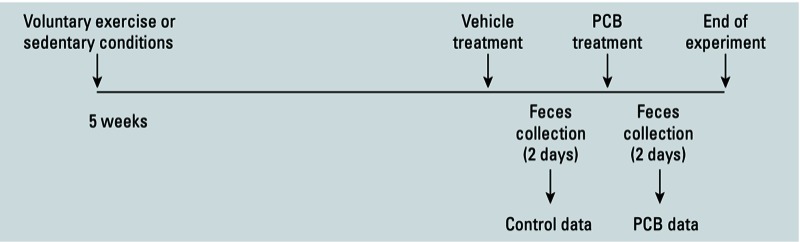
Experimental design indicating treatment and sampling times.

A diagram of the study design is shown in Figure 1. At the end of the exercise period, mice were administered tocopherol-stripped safflower oil (5 mL/kg) to allow them to adjust to the vehicle. Feces were collected for 2 days (allowing collection of a sufficient amount of sample), and samples were analyzed for control/background microbiome data. Mice were then treated with the PCB mixture, and feces were again collected for 2 days. To minimize the influence of individual variations on the gut microbiome, each mouse served as its own control for PCB treatment.

*Analysis of the gut microbiome*. All analyses were performed using PhyloChip Arrays (Second Genome Inc., San Bruno, CA). The GenBank accession numbers were used as identification and classification of bacterial operational taxonomic units (OTUs) based on their 16S rRNA sequences. Bacterial 16S rRNAs were amplified with the primer sets 27F.1 (5´-AGRG​TTTG​ATCM​TGGC​TCAG-3´) and 1492R.jgi (5´-GGTT​ACCT​TGTT​ACGA​CTT-3´). Gene amplification was accomplished by polymerase chain reaction (PCR; 35 cycles) as follows: denature step at 95°C for 30 sec, annealing step at 50°C for 30 sec, and extension step at 72°C for 2 min using the TaKaRa Ex Taq system (Takara Bio Inc., Japan). For each sample, amplified products were concentrated using a centrifuge filtration method and quantified by electrophoresis using an Agilent 2100 Bioanalyzer (Agilent Technologies, Santa Clara, CA). PhyloChip Control Mix (Second Genome Inc.) was added to each amplified product. The combined PCR products and controls were fragmented, biotin labeled, and hybridized to the PhyloChip Array (version G3). PhyloChip arrays were washed, stained, and scanned using a GeneArray scanner (Affymetrix, Cleveland, OH). Each scan was captured using the GeneChip Microarray Analysis Suite (Affymetrix). Hybridization scores and the fluorescence intensity for each taxon were calculated as a trimmed average, with maximum and minimum values removed before averaging.

*Statistical analysis*. Data processing and multivariate statistical analyses were performed using the PhyCA-Stats analysis software package (Second Genome Inc.). Probe intensities were background subtracted and scaled to values for PhyloChip Control Mix. Subsequently, hybridization scores were calculated as log_2_(mean probe fluorescence intensity) × 1,000. OTUs were defined by high 16S rRNA gene sequence similarity, with most demonstrating > 99% intra-OTU concordance. We determined presence/absence of an OTU by a threshold based on perfect match and mismatch intensities of multiple probes per probe set (Hazen et al 2010). Before classification analysis, we performed data reduction via multiple filtering steps as described by Hazen et al. (2010). Taxa-sample intersections were then calculated using abundance (AT) and binary matrices (BT). Pairwise BT and AT dissimilarity scores were computed using the Unifrac distance metric (Lozupone et al. 2006) and weighted Unifrac distance metric, respectively; the weighted Unifrac metric considers OTU abundance in addition to phylogenetic distance between OTUs. We used hierarchical clustering via average-neighbor (HC-AN) and principal coordinate analysis (PCoA) to graphically summarize intersample relationships according to AT and BT dissimilarity scores. Unsupervised classification using the nearest shrunken centroid method (Tibshirani et al. 2002) as implemented in Prediction Analysis of Microarrays (PAM; http://www-stat.stanford.edu/~tibs/PAM/) was used to identify OTUs with the most significant differences in abundance between comparison groups. We used a randomization/Monte Carlo permutation–based test (Adonis test) to determine significant differences between discrete and continuous variables. Finally, we employed Welch’s test and Student’s *t*-test for statistical significance, with *p* < 0.05 considered significant.

## Results

*PCB exposure decreases the abundance of the gut microbiota*. We first analyzed the effects of PCB exposure on the gut microbiome in sedentary mice. The Welch’s test revealed that exposure to the PCB mixture significantly (*p* < 0.05 by Student’s *t*-test) altered the abundance of 1,223 bacterial taxa in these mice (1,133 taxa had decreased abundance and 90 taxa had increased abundance). As a result of these changes, the overall abundance of bacteria significantly diminished (by 2.2%) in PCB-exposed mice. Table 1 lists bacterial taxa with the greatest decrease in abundance (4.0- to 5.6-fold) after PCB treatment. These taxa belong primarily to phylum Proteobacteria, but the classes and families are diverse. The group of bacterial taxa with increased abundance after PCB treatment was relatively modest, and the changes did not exceed 2-fold; the bacterial taxa of these with the highest increases in abundance are listed in Table 2. They belong to several difference phyla, including Bacteriodetes, Actinobacteria, Verrucomicrobia, and Firmicutes.

**Table 1:** The 10 bacterial taxa with the greatest decrease in abundance following exposure of sedentary mice to the PCB mixture.

Phylum	Class	Order	Family	Species	GenBank accession ID	Fold change
Proteobacteria	Gammaproteobacteria	Pseudomonadales	Pseudomonadaceae	*Pseudomonas plecoglossicida *strain CGMCC 2093	EF645247	–5.6
Proteobacteria	Gammaproteobacteria	Pseudomonadales	Pseudomonadaceae		FJ901066	–4.8
Proteobacteria	Betaproteobacteria	Burkholderiales	Comamonadaceae		GQ007353	–4.4
Proteobacteria	Betaproteobacteria	Burkholderiales	Comamonadaceae		GQ108141	–4.4
Proteobacteria	Gammaproteobacteria	Pseudomonadales	Pseudomonadaceae	*Pseudomonas plecoglossicida *strain R18	DQ095882	–4.3
Proteobacteria	Betaproteobacteria	Burkholderiales	Comamonadaceae		GQ008724	–4.3
Proteobacteria	Betaproteobacteria	Burkholderiales	Comamonadaceae		GQ100754	–4.1
Proteobacteria	Gammaproteobacteria	Pseudomonadales	Pseudomonadaceae	*Pseudomonas putida *strain SRI156	EU826028	–4.0
Firmicutes	Bacilli	Lactobacillales	Streptococcaceae	*Streptococcus infantis*	GQ077246	–4.0
Proteobacteria	Betaproteobacteria	Burkholderiales	Comamonadaceae		EF520494	–4.0
Fold change represents the relative bacterial abundance in PCB-exposed mice compared with vehicle-treated controls; values were calculated from the fluorescence intensities derived from hybridization scores.						

**Table 2:** The 10 bacterial taxa with the greatest increase in abundance following exposure of sedentary mice to the PCB mixture.

Phylum	Class	Order	Family	Species	GenBank accession ID	Fold change
Bacteroidetes	Sphingobacteria	Sphingobacteriales	Saprospiraceae	*Candidatus aquirestis calciphila*	AY863078	1.9
Actinobacteria	Actinobacteria	Actinomycetales	Corynebacteriaceae		GQ083745	1.7
Verrucomicrobia	Verrucomicrobiae	Verrucomicrobiales	Verrucomicrobiaceae		FM242339	1.7
Firmicutes	Bacilli	Bacillales	Staphylococcaceae	*Staphylococcus epidermidis*	GQ087061	1.7
Bacteriodetes	Bacteroidia	Bacteroidales	Bacteroidaceae	*Bacteroides thetaiotaomicron *strain**8669	AY895200	1.6
Cyanobacteria	Chloroplast	Chlorophyta	Ulvophyceae		FJ203465	1.6
Actinobacteria	Actinobacteria	Actinomycetales	Corynebacteriaceae		GQ039749	1.5
Bacteriodetes	Bacteroidia	Bacteroidales	Porphyromonadaceae		AB231049	1.5
Actinobacteria	Actinobacteria	Actinomycetale	Corynebacteriaceae	*Tropheryma whipplei*	AF190688	1.5
Actinobacteria	Actinobacteria	Actinomycetales	Corynebacteriaceae		GQ055950	1.5
Fold change represents the relative bacterial abundance in PCB-exposed mice compared with vehicle-treated controls; values were calculated from the fluorescence intensities derived from hybridization scores.						

Exposure to the PCB mixture did not alter biodiversity of the gut microbiome; all 11,229 taxa were detected in at least one sample from sedentary mice. After PCB exposure, the number of taxa dropped to 10,798, but the change was not statistically significant.

**Figure 2:**
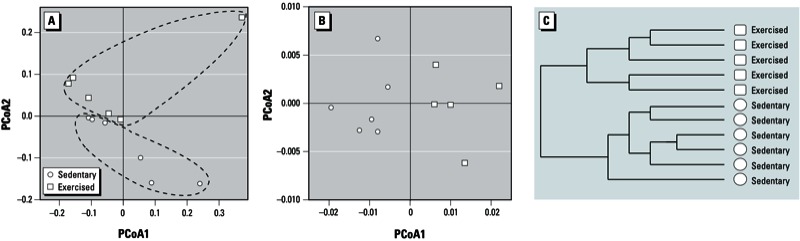
Exercise alters the structure and composition of the gut microbiome. (*A*) PCoA based on unweighted Unifrac distance between exercised and sedentary mice (PCoA1, 33% of variation; PCoA2, 15% of variation). (*B*) PCoA and (*C*) HC‑AN analysis based on weighted Unifrac distance between exercised and sedentary mice of the 2,510 taxa with significant abundance differences across at least one of the categories (PCoA1, 84% of variation; PCoA2, 6% of variation).

*Exercise alters the composition of the gut microbiome*. As analyzed by weighted Unifrac distance, the structure of the gut microbiome of the exercised mice was significantly different that of the sedentary mice (Adonis test, *p* < 0.05). PCoA of unweighted Unifrac distance with given presence/absence metrics also showed prominent categorization of the composition of the gut microbiome between the exercised and sedentary mice (Figure 2A).

Among detected bacterial taxa, 93 were present exclusively in either exercised or sedentary mice. Specifically, 67 taxa were detected only in the exercised mice [see Supplemental Material, Table S1 (http://dx.doi.org/10.1289/ehp.1306534)], and 26 taxa were unique to sedentary mice (see Supplemental Material, Table S2).

A group of 2,510 taxa showed differences in abundance between the exercised and sedentary mice. These taxa were then analyzed by PCoA with weighted Unifrac distance, which indicated significant differences in the composition of the microbial communities between the exercised and sedentary mice (Figure 2B). HC-AN analysis based on weighted Unifrac distance confirmed a shift of the composition of the gut microbiome related to physical activity (exercised vs. sedentary mice) (Figure 2C).

Further examination using the PAM identified 10 taxa with substantially different abundance between the exercised and sedentary mice (Table 3). The taxa that were more abundant in the exercised group were in phylum Firmicutes, class Bacilli, and most of these were in order Lactobacillales. The taxa that were decreased in the exercised group belonged to phyla Tenericutes, Bacteroidetes, and Firmicutes. The most striking change in exercised mice was a decrease in Erysipelotrichaceae bacterium C11_K211 from phylum Tenericutes, which decreased dramatically in exercised compared with sedentary mice.

**Table 3:** Prediction analysis for microarrays (PAM)-selected distinctive bacterial taxa presenting differentially in the mouse gut microbiome of excercised versus sedentary mice.

Phylum	Class	Order	Family	Species	GenBank accession ID	Fold change
Firmicutes	Bacilli	Lactobacillales	Enterococcaceae	*Enterococcus faecium*	EF533987	24.1
Firmicutes	Bacilli	Lactobacillales	Enterococcaceae	*Enterococcus faecium*	AY692451	15.7
Firmicutes	Bacilli	Bacillales	Staphylococcaceae	*Staphylococcus gallinarum*	DQ350835	12.1
Firmicutes	Bacilli	Lactobacillales	Enterococcaceae	*Escherichia coli O157:H7*	FJ675223	7.6
Firmicutes	Bacilli	Lactobacillales	Enterococcaceae	*Enterococcus faecium*	FJ378658	7.4
Firmicutes	Bacilli	Lactobacillales	Streptococcaceae	*Streptococcus pseudopneumoniae*	GQ000464	8.7
Firmicutes	Bacilli	Bacillales	Bacillaceae	*Bacillus trypoxylicola*	AB434284	5.8
Tenericutes	Erysipelotrichi	Erysipelotrichales	Erysipelotrichaceae	*C11_K211*	DQ015346	–361
Firmicutes	Clostridia	Clostridiales	Ruminococcaceae		EU453081	–6.4
Bacteroidetes	Bacteroidia	Bacteroidales	Bacteroidaceae	*Bacteroides clarus*	AB490801	–8.6
Fold change represents the relative bacterial abundance in exercised compared with sedentary mice; values were calculated from the fluorescence intensities derived from hybridization scores.						

**Figure 3:**
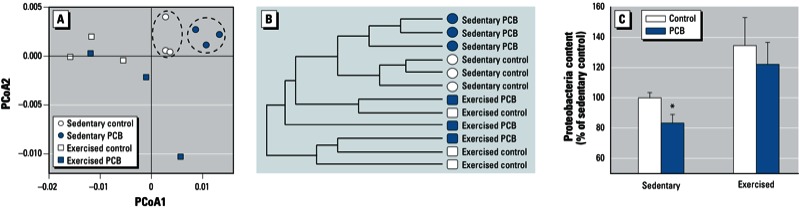
Exercise prevents PCB-induced alterations of the gut microbiome. (*A*) PCoA and (*B*) HC‑AN analysis based on weighted Unifrac distance of the 1,568 taxa with significant differences in abundance in at least one of the categories (PCoA1, 71% of variation; PCoA2, 11% of variation). (*C*) Proteobacteria content in vehicle-treated control and PCB-treated sedentary and exercised mice; values are mean ± SE of pooled taxa in phylum Proteobacteria. *p < 0.05 compared with controls.

*Exercise attenuates PCB-induced alterations of gut microbiome composition*. Comparison of the gut microbiome among all experimental groups (sedentary and exercised mice with or without PCB exposure) identified 1,568 bacterial taxa that were differentially abundant in at least one of these groups. Analysis of these taxa for dissimilarity between the groups using PCoA with weighted Unifrac indicated significant differences between the composition of the gut microbiome before and after PCB exposure in sedentary mice. Importantly, exercise altered PCB-mediated effects on the gut microbiome, as indicated by a loss of bacterial clustering (Figure 3A). This phenomenon was subsequently confirmed by HC-AN analysis (Figure 3B).

We detected 10,799 taxa in sedentary mice exposed to the PCB mixture, compared with 13,383 taxa in the exercised mice treated with PCBs. Although there appeared to be a tendency toward increased biodiversity in the exercise-plus-PCB group, these changes were not significant. In contrast, the abundance of bacterial species was elevated by 2.9% (Student’s *t*-test, *p* < 0.05) in PCB-treated exercised mice as compared PCB-treated sedentary mice, providing additional evidence that exercise can protect against PCB-mediated alterations in the gut microbiota. As shown in Figure 3C, exercise appeared to prevent a PCB-induced decrease in abundance of Proteobacteria in sedentary mice (Figure 3C).

## Discussion

In view of recent evidence indicating that gut bacteria can be involved in the preabsorptive metabolism of heavy metals and organic chemicals (Lapanje et al. 2007; Liebert et al. 1997; Pinyayev et al. 2011), the gut microbiome has been proposed to play a role in the assessment of health risks associated with environmental chemicals (Betts 2011; Snedeker and Hay 2012). Therefore, our demonstration that short-term exposure to an environmentally relevant PCB mixture resulted in profound changes in the gut microbiome in mice is highly significant. The most striking change in the intestinal microbial profiles was a decrease in the overall abundance of bacterial species. Although these results are the first to show effects of PCBs on the gut microbiome, the decrease in bacterial abundance we observed corresponds with the finding that PCB-contaminated soil is characterized by a shift in structure and abundance of bacterial community (Petric et al. 2011). Incubation of soil slurries with higher-chlorinated PCB congeners (e.g., PCB28, PCB77, Aroclor 1242) has been reported to result in lower bacterial numbers (Correa et al. 2010). Even though the gut and soil provide completely different bacterial environments, PCB exposure appears to elicit environmental stress on the structure and composition of bacterial communities that results in diminished bacterial abundance. In the present study, these changes selectively affected bacterial phylotypes in phylum Proteobacteria. Such changes may be most relevant to immune functions of the host, because the gut microbiome has been shown to play important roles in mucosal immunity and interactions with intestinal and colonic epithelial cells, dendritic cells, and T and B immune cells. Microbiota composition has functional effects on T-effector- and T-regulatory-cell balance, immune responsiveness, and homeostasis (Kelly and Mulder 2012). Thus, it is likely that alterations of the gut microbiota compromise a novel mechanism leading to immunological alterations, which develop in response to exposure to PCBs (Maule et al. 2005; Weisglas-Kuperus et al. 2004). In addition, alterations of the gut microbiome can affect PCB-induced disruption of the intestinal barrier and translocation of lipopolysaccharides into the blood stream (Choi JJ et al. 2012; Choi YJ et al. 2010). In the present study, we observed no statistical differences in bacterial community structure in the exercised mice before and after PCB treatment. Thus, exercise provided protection against PCB-induced changes in the gut microbiome. In particular, exercise prevented a PCB-induced decrease in abundance of Proteobacteria, which was observed in sedentary mice.

Although the data we reported here are novel, effects of physical activity on several other aspects of gut physiology (e.g., peristalsis; Song et al. 2012) and pathology have been reported. For example, exercise was demonstrated to decrease the risk of developing several intestinal diseases, including colon cancer (Friedenreich et al. 2006), inflammatory bowel disease and irritable bowel syndrome (Lustyk et al. 2001), and other disorders that are accompanied by changes in the gut microbiome (De Hertogh et al. 2012; Nelson et al. 2011; Walker et al. 2011).

The mechanisms of exercise-mediated changes in gut ecology are not known; however, they are likely to be mediated by altering the host factors that influence the intestinal microenvironment. For example, physical activity has been reported to increase excretion of primary bile acids to the gastrointestinal tract (Meissner et al. 2011) and to suppress the formation of secondary bile acids (Hagio et al. 2010). The primary bile acids, such as cholic, deoxycholic, or chenodeoxycholic acids, have established antimicrobial activity, which is mediated by the reduction in internal pH levels of bacteria, dissipation of their transmembrane electrical potential, and disturbances of membrane integrity, leading to leakage of ions and cell death (Kurdi et al. 2006). In support of this hypothesis, Islam et al. (2011) demonstrated that cholic acid induced substantial changes in the cecal microbiome composition by stimulating the growth of Firmicutes at the expense of Bacteroidetes and outgrowth of several bacteria in the classes Clostridia and Erysipelotrichi. Thus, the antimicrobial activity of the bile acids may elicit selective pressure on the bacterial communities in exercised mice, leading to a shift of the gut microbiome structure as observed in the present study.

Short-chain fatty acids (SCFAs) may be another factor that regulates the gut microbiome in response to exercise. Indeed, Matsumoto et al. (2008) showed that rats that participated in voluntary running exercise had increased butyrate concentration in the cecum compared with sedentary rats. These authors directly linked this effect to the beneficial effect of exercise on the gut microbiota and the development of gastrointestinal disorders. SCFAs (e.g. butyrate and acetate) increase colonic epithelial cell proliferation and decrease the risk of colorectal cancer. Their influence on the composition of microbial environment has been linked to a decreased pH in the gut (Wong et al. 2006). Nevertheless, compared with the results of the present study, the effects of butyrate infusion on the rumen microbiome in cows was relatively minor because only 19 genera and 43 bacterial taxa were significantly affected in response to butyrate (Li et al. 2012); these data suggest that this SCFA may be only one of several factors involved in exercise-mediated changes in the gut microbiome. Treatment with SCFAs may also affect host-related intestinal factors because butyrate promotes cell differentiation and cell-cycle arrest, inhibits the enzyme histone deacetylase, and decreases the transformation of primary to secondary bile acids as a result of colonic acidification (Wong et al. 2006).

Finally, exercise may influence the composition of the gut microbiome by altering the intestinal immune system. Viloria et al. (2011) observed that physical activity increased expression of IgA and cytokines such as interleukin-6 and tumor necrosis factor-α. These changes in the intestinal immune system may lead to secondary alternations of the host–bacterial interaction and induce selective pressure on bacterial selection.

The development of chronic diseases related to the exposure to environmental toxicants are associated with age, and the benefits of exercise are also being emphasized in older individuals; therefore, we used aged mice (11–13 months of age) in the present study. Older mice tend to have higher body mass than younger animals. In fact, the average body weight of sedentary mice in the present study was 46.8 ± 1.4 g, and body weight of exercised mice was approximately 30% lower. Recent evidence from Ley et al. (2005) indicated a strong association of the intestinal microbiome with the development of obesity. Genetically obese *ob/ob* mice were characterized by a major decrease in the abundance of Bacteroidetes and an increase in Firmicutes compared with lean *ob/+* wild-type littermates and lean *ob/+* mothers fed the same diets. Similar changes were observed in wild-type mice fed a high fat/high polysaccharide diet (Turnbaugh et al. 2008) and in obese humans (Ley et al. 2005). An increase in Firmicutes (such as Lactobacilli) and a decrease in Bacteroidetes have been confirmed in obese humans (Armougom et al. 2009). In another study, overweight pregnant patients had reduced abundance of Bifidobacteria and Bacteroidetes and increased abundance of selected Firmicutes (e.g., *Staphylococcus*) and Proteobacteria (e.g., Enterobacteriaceae) (Santacruz et al. 2010). In line with these reports, it is relevant that we detected an increased abundance of several Firmicutes, primarily Enterococcaceae (e.g., *Enterococcus faecium*), in the exercised mice.

Although Enterococci are commensal bacteria, they are also important nosocomial pathogens that cause bacteremia, endocarditis, and other infections in humans. Some strains are resistant to multiple antibiotics and possess virulence factors, such as adhesins, invasins, pili, and haemolysin (Willems et al. 2011). Nevertheless, *E. faecium* isolates from clinical outbreaks are different strains than *E. faecium* from animals, food, and humans in the community (Franz et al. 2011). In fact, several enterococci, including *E. faecium* strains, are used as probiotics in the form of pharmaceutical preparations. They are administered to treat diarrhea, antibiotic-associated diarrhea, and irritable bowel syndrome; to lower cholesterol levels; or to improve host immunity (Franz et al. 2011).

The most striking change we found in the gut microbiome of exercised mice was a > 300-fold decrease in the abundance of Erysipelotrichaceae (Table 3). This family plays an important role in metabolic disorders and energy metabolism (Chen et al. 2012). Chen et al. (2012) reported that Erysipelotrichaceae were enriched in obese humans and mice, as well as in mice fed a high-fat diet, and noted that the abundance of Erysipelotrichaceae was also increased in patients with colorectal cancer. Thus, our data are consistent because we observed a decrease in the number of Erysipelotrichaceae in exercised mice that had lost a substantial amount of body weight; this is associated with the role of Erysipelotrichaceae in energy production and adiposity (Claus et al. 2011; Goodman et al. 2011; Zhang et al. 2009).

## Conclusions

Here we demonstrate that oral exposure to a mixture of environmentally relevant PCB congeners significantly altered the abundance of the gut microbiome by decreasing the levels of Proteobacteria. These results suggest that the gut microbiome may be one of the primary targets of PCB-induced toxicity in subjects exposed orally to these environmental toxicants. We observed that PCB-induced alterations of the gut microbiome were attenuated by voluntary exercise.

## Supplemental Material

(553 KB) PDF
