# Supplementary material for: Exercise Attenuates PCB-Induced Changes in the Mouse Gut Microbiome
Source: Environ Health Perspect. 2013 Apr 26;121(6):725–30. doi: 10.1289/ehp.1306534 (PMC3672930; doi:10.1289/ehp.1306534)
Supplement: (553 KB) PDF [file ehp.1306534.s001.pdf]

Supplemental Material

Exercise Attenuates PCB-Induced Changes in the Mouse Gut Microbiome

Jeong June Choi, Sung Yong Eum, Evadnie Rampersaud, Sylvia Daunert, Maria T Abreu, and Michal Toborek

TABLE OF CONTENTS

Table S1. Bacterial taxa present in exercised mice only **2**

Table S2. Bacterial taxa present in sedentary mice only **6**

Supplemental Material, Table S1. Bacterial taxa present in exercised mice only

Phylum	Class	Order	Family	Species	GenBank Accession ID
Firmicutes	Bacilli	Lactobacillales	*Enterococcaceae*	*Enterococcus faecium*	EF533987
Firmicutes	Bacilli	Lactobacillales	*Enterococcaceae*	*Enterococcus faecium*	FJ378668
Firmicutes	Bacilli	Lactobacillales	*Enterococcaceae*	*Enterococcus faecium*	FJ378683
Firmicutes	Bacilli	Lactobacillales	*Enterococcaceae*	*Enterococcus faecium*	FJ378661
Firmicutes	Bacilli	Lactobacillales	*Enterococcaceae*	*Enterococcus faecium*	FJ378674
Firmicutes	Bacilli	Lactobacillales	*Enterococcaceae*	*Enterococcus faecium*	Y18294
Firmicutes	Bacilli	Lactobacillales	*Enterococcaceae*	*Enterococcus faecium*	AJ874342
Firmicutes	Bacilli	Lactobacillales	*Enterococcaceae*	*Enterococcus faecium*	AY057055
Firmicutes	Bacilli	Lactobacillales	*Enterococcaceae*	*Enterococcus faecium*	AM157434
Firmicutes	Bacilli	Lactobacillales	*Enterococcaceae*	*Enterococcus faecium*	EU878169
Firmicutes	Bacilli	Lactobacillales	*Enterococcaceae*	*Enterococcus faecium*	AY653231
Firmicutes	Bacilli	Lactobacillales	*Enterococcaceae*	*Enterococcus faecium*	EU807757
Firmicutes	Bacilli	Lactobacillales	*Enterococcaceae*	*Enterococcus faecalis*	FJ378658
Firmicutes	Bacilli	Lactobacillales	*Enterococcaceae*	*Enterococcus faecium*	AJ420800
Firmicutes	Bacilli	Lactobacillales	*Enterococcaceae*	*Enterococcus faecium*	FJ378686
Firmicutes	Bacilli	Lactobacillales	*Enterococcaceae*	*Enterococcus gallinarum*	GQ337019
Firmicutes	Bacilli	Lactobacillales	*Enterococcaceae*	*Enterococcus faecium*	EU878170
Firmicutes	Bacilli	Lactobacillales	*Enterococcaceae*	*Enterococcus faecium*	EU428012

**Phylum**	**Class**	**Order**	**Family**	**Species**	**GenBank Accession ID**
Firmicutes	Bacilli	Lactobacillales	*Enterococcaceae*	*Enterococcus faecium*	FJ378689
Firmicutes	Bacilli	Lactobacillales	*Enterococcaceae*	*Enterococcus faecium*	FJ378697
Firmicutes	Bacilli	Lactobacillales	*Enterococcaceae*	*Enterococcus faecium*	NZ_ACIY01000209
Firmicutes	Bacilli	Lactobacillales	*Enterococcaceae*	*Enterococcus faecium*	FJ378673
Firmicutes	Bacilli	Lactobacillales	*Enterococcaceae*	*Enterococcus faecium*	AF070223
Firmicutes	Bacilli	Lactobacillales	*Enterococcaceae*	*Enterococcus faecium*	FJ378670
Firmicutes	Bacilli	Lactobacillales	*Enterococcaceae*	*Enterococcus faecium*	EU003448
Firmicutes	Bacilli	Lactobacillales	*Enterococcaceae*	*Enterococcus faecium*	FJ378693
Firmicutes	Bacilli	Lactobacillales	*Enterococcaceae*	*Enterococcus lactis*	FJ015055
Firmicutes	Bacilli	Lactobacillales	*Enterococcaceae*	*Enterococcus faecium*	AY971749
Firmicutes	Bacilli	Bacillales	*Bacillaceae*	*Bacillus subtilis*	EF990557
Firmicutes	Bacilli	Lactobacillales	*Enterococcaceae*	*Enterococcus casseliflavus*	X76177
Firmicutes	Bacilli	Lactobacillales	*Streptococcaceae*		AY005040
Proteobacteria	Gammaproteobacteria	Pseudomonadales	*Pseudomonadaceae*		EU538172
Firmicutes	Bacilli	Lactobacillales	*Aerococcaceae*	*Aerococcus urinae*	U64457
Firmicutes	Bacilli	Lactobacillales	*Enterococcaceae*	*Enterococcus hirae*	FJ851687
Firmicutes	Bacilli	Lactobacillales	*Enterococcaceae*	*Enterococcus lactis*	EU717965
Bacteroidetes	Bacteroidia	Bacteroidales	*Bacteroidaceae*		EU763339
Firmicutes	Clostridia	Clostridiales	*Lachnospiraceae*		DQ015047

**Phylum**	**Class**	**Order**	**Family**	**Species**	**GenBank Accession ID**
Actinobacteria	Actinobacteria	Actinomycetales	*Micromonosporaceae*	*Catenuloplanes niger*	FJ715940
Firmicutes	Bacilli	Lactobacillales	*Enterococcaceae*	*Enterococcus villorum*	AF335596
Firmicutes	Bacilli	Lactobacillales	*Enterococcaceae*		GQ155230
Firmicutes	Bacilli	Lactobacillales	*Aerococcaceae*	*Aerococcus viridans*	AY707773
Bacteroidetes	Bacteroidia	Bacteroidales	*Bacteroidaceae*		EU768032
Bacteroidetes	Bacteroidia	Bacteroidales	*RikenellaceaeII*	*Bacteroides nordii*	EU887841
Firmicutes	Bacilli	Lactobacillales	*Enterococcaceae*	*Enterococcus canintestini*	GQ337018
Firmicutes	Clostridia	Clostridiales	*Lachnospiraceae*		DQ795656
Firmicutes	Clostridia	Clostridiales	*Lachnospiraceae*	*Eubacterium rectale*	FJ367978
Proteobacteria	Gammaproteobacteria	Enterobacteriales	*Enterobacteriaceae*	*Citrobacter freundii*	AY163805
Bacteroidetes	Bacteroidia	Bacteroidales	*Bacteroidaceae*		EU767916
Firmicutes	Clostridia	Clostridiales	*Lachnospiraceae*		EU452598
Firmicutes	Bacilli	Bacillales	*Bacillaceae*	*Bacillus subtilis*	FJ544352
Bacteroidetes	Bacteroidia	Bacteroidales	*Bacteroidaceae*		EU762810
Firmicutes	Bacilli	Lactobacillales	*Enterococcaceae*	*Enterococcus hirae*	AJ301834
Bacteroidetes	Bacteroidia	Bacteroidales	*Bacteroidaceae*		EU763101
Nitrospirae	Nitrospira	Nitrospirales	*Thermodesulfovibrionaceae*		FJ638591
Firmicutes	Bacilli	Bacillales	*Bacillaceae*	*Geobacillus thermoleovoran*	AY450926
Proteobacteria	Betaproteobacteria	Burkholderiales	*Aquabacteriaceae*		GQ102733

**Phylum**	**Class**	**Order**	**Family**	**Species**	**GenBank Accession ID**
Actinobacteria	Actinobacteria	Actinomycetales	*Corynebacteriaceae*		GQ035028
Firmicutes	Clostridia	Clostridiales	*Lachnospiraceae*	*Clostridium nexile*	X73443
Firmicutes	Clostridia	Clostridiales	*Ruminococcaceae*		FJ370347
Bacteroidetes	Bacteroidia	Bacteroidales	*RikenellaceaeII*		EU771159
Bacteroidetes	Flavobacteria	Flavobacteriales	*Flavobacteriaceae*		GQ098103
Firmicutes	Clostridia	Clostridiales	*Lachnospiraceae*		AY982924
Firmicutes	Clostridia	Clostridiales	*Lachnospiraceae*	*Eubacterium rectale*	FJ684683
Tenericutes	Mollicutes	RF39	unclassified		EU842453
Firmicutes	Bacilli	Bacillales	*Planococcaceae*	*Ureibacillus*	AB170018
Bacteroidetes	Bacteroidia	Bacteroidales	*RikenellaceaeII*		EU504144
Bacteroidetes	Sphingobacteria	Sphingobacteriales	*Saprospiraceae*		GU230422

Supplemental Material, Table S2. Bacterial taxa present in sedentary mice only

Phylum	Class	Order	Family	Species	GenBank Accession ID
Tenericutes	Erysipelotrichi	Erysipelotrichales	*Erysipelotrichaceae*	*C11_K211*	DQ015346
Firmicutes	Clostridia	Clostridiales	*Ruminococcaceae*		EU453704
Firmicutes	Clostridia	Clostridiales	*Lachnospiraceae*		EF099589
Firmicutes	Bacilli	Bacillales	*Bacillaceae*	*Bacillus halodurans*	EF113315
Firmicutes	Clostridia	Clostridiales	*Lachnospiraceae*		AB494756
Firmicutes	Clostridia	Clostridiales	*Lachnospiraceae*		EU453854
Firmicutes	Clostridia	Clostridiales	*Lachnospiraceae*		FJ674265
Firmicutes	Clostridia	Clostridiales	*Lachnospiraceae*		EU472163
Bacteroidetes	Bacteroidia	Bacteroidales	*RikenellaceaeII*		CU922957
Firmicutes	Clostridia	Clostridiales	*Ruminococcaceae*		EF399615
Proteobacteria	Betaproteobacteria	Burkholderiales	*Aquabacteriaceae*		GQ115257
Proteobacteria	Betaproteobacteria	Burkholderiales	*Aquabacteriaceae*		GQ037260
Proteobacteria	Betaproteobacteria	Burkholderiales	*Aquabacteriaceae*		GQ099604
Firmicutes	Clostridia	Clostridiales	*Ruminococcaceae*		FJ682721
Proteobacteria	Gammaproteobacteria	Pseudomonadales	*Pseudomonadaceae*		EF620447
Firmicutes	Clostridia	Clostridiales	*Ruminococcaceae*		FJ674232
Proteobacteria	Gammaproteobacteria	Oceanospirillales	*Oceanospirillaceae*		FJ403057
Firmicutes	Clostridia	Clostridiales	*Ruminococcaceae*		AB185773

**Phylum**	**Class**	**Order**	**Family**	**Species**	**GenBank Accession ID**
Firmicutes	Clostridia	Clostridiales	*Ruminococcaceae*		FJ371863
Firmicutes	Bacilli	Exiguobacterales	*Exiguobacteraceae*		FJ821599
Proteobacteria	Betaproteobacteria	Burkholderiales	*Aquabacteriaceae*		GQ071248
Firmicutes	Bacilli	Bacillales	*Bacillaceae*		AB245377
Firmicutes	Clostridia	Clostridiales	*Ruminococcaceae*		EU460195
Firmicutes	Clostridia	Clostridiales	*Lachnospiraceae*		DQ800645
Firmicutes	Clostridia	Clostridiales	*Lachnospiraceae*		AJ408971
Bacteroidetes	Bacteroidia	Bacteroidales	*Rikenellaceae*		AY993090
